# Hydrogel Composite Magnetic Scaffolds: Toward Cell-Free In Situ Bone Tissue Engineering

**DOI:** 10.1021/acsabm.3c00732

**Published:** 2023-12-18

**Authors:** Jingyi Xue, Neelam Gurav, Sherif Elsharkawy, Sanjukta Deb

**Affiliations:** Faculty of Dentistry, Oral and Craniofacial Sciences, King’s College London, London SE1 9RT, United Kingdom

**Keywords:** magnetic nanoparticles, hydrogel composites, bone tissue engineering, scaffolds, biomineralization, poly(vinyl alcohol)

## Abstract

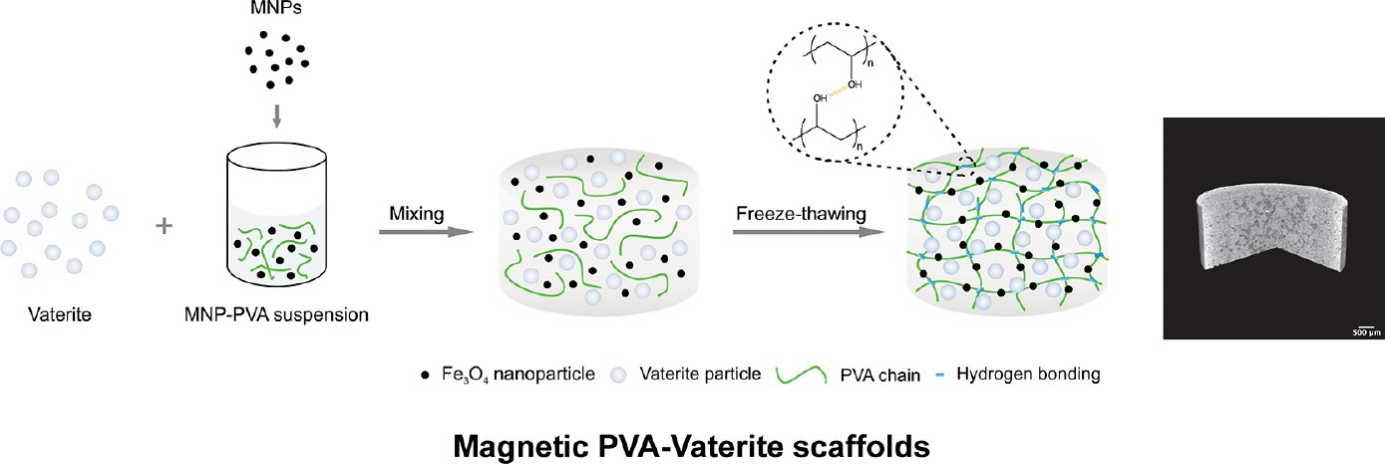

Reconstruction of critical sized bone defects in the oral and maxillofacial region continues to be clinically challenging despite the significant development of osteo-regenerative materials. Among 3D biomaterials, hydrogels and hydrogel composites have been explored for bone regeneration, however, their inferior clinical performance in comparison to autografts is mainly attributed to variable rates of degradation and lack of vascularization. In this study, we report hydrogel composite magnetic scaffolds formed from calcium carbonate, poly(vinyl alcohol) (PVA), and magnetic nanoparticles (MNPs), using PVA as matrix and calcium carbonate particles in vaterite phase as filler, to enhance the cross-linking of matrix and porosity with MNPs that can target and regulate cell signaling pathways to control cell behavior and improve the osteogenic and angiogenic potential. The physical and mechanical properties were evaluated, and cytocompatibility was investigated by culturing human osteoblast-like cells onto the scaffolds. The vaterite phase due to its higher solubility in comparison to calcium phosphates, combined with the freezing–thawing process of PVA, yielded porous scaffolds that exhibited adequate thermal stability, favorable water-absorbing capacity, excellent mineralization ability, and cytocompatibility. An increasing concentration from 1, 3, and 6 wt % MNPs in the scaffolds showed a statistically significant increase in compressive strength and modulus of the dry specimens that exhibited brittle fracture. However, the hydrated specimens were compressible and showed a slight decrease in compressive strength with 6% MNPs, although this value was higher compared to that of the scaffolds with no MNPs.

## 1 Introduction

Management of large bone defects remains clinically challenging, and a major impediment is insufficient vascularization of the graft material to effect successful bone healing. The current gold standard for bone reconstruction using autografts shows good clinical outcomes but is associated with significant second site morbidity, and their limited availability necessitates the use of synthetic bone substitutes (SBS).^1^ Current bone substitutes used clinically are largely based on calcium phosphates that have certain desirable characteristics, however, most are brittle, exhibit variable resorption rates, are not intrinsically osteoinductive with limited angiogenic potential, and tend to present variable clinical outcomes.^2^ Tissue engineering of bone provides an alternative approach that can introduce an exogenous three-dimensional scaffold with a morphologically controlled tailored interconnected porous structure that encourages cells to grow and proliferate. It has now become increasingly evident that hierarchical integration of scaffolds that support both osteogenic and angiogenic growth is a key factor for successful bone regeneration.^3^

Hydrogels have been explored as matrices for tissue engineering since they can closely mimic the extra cellular matrix, their properties can be tailored to suit mechanical requirements, and they can encapsulate cells and bioactive molecules.^4^ However, to minimize the complications that arise with inclusion of biological factors or cells in scaffolds, current directions are focused on developing smart composite substrates that can utilize endogenous cells for in situ tissue bone regeneration.^3^

Magnetic nanoparticles (MNPs) with magneto-mechanical forces acting as intracellular and extracellular signal control have been used to accelerate cell growth, organize vascular networks, and develop tissues.^5^ Superparamagnetic iron oxide nanoparticles (SPIONs) generate an intrinsic magnetic field, and each magnetic particle is considered as a single magnetic domain, able to provide a magnetic field at a nanoscale level. Previous studies have shown that MNPs in composite scaffolds can be internalized by osteoblasts and vascular endothelial cells, leading to elevated osteogenic and angiogenic performance.^6,7^ In addition, magnetic nanoparticles increase cell adhesion by providing a greater surface area, higher roughness, and hydrophilicity in scaffolds.^8^ The strong interaction between magnetic nanoparticles and the matrix also increases the mechanical strength of scaffolds.^9^ Among various magnetic nanoparticles, magnetite (Fe_3_O_4_) nanoparticles have been shown to exhibit good biocompatibility and unique magnetic properties that stimulate osteogenic differentiation and promote growth factor expression in mesenchymal stem cells (MSCs).^10^ Magnetite nanoparticles can rapidly reach saturation magnetization when exposed to a modest external magnetic field, which can be easily altered and aligned along the preferred direction, allowing a wide range of biomedical applications like magneto-mechanical forces for controlling cell signaling. Thus, the addition of SPIONs to scaffolds is expected to yield not only mechanically strong scaffolds but also possess bone-forming capacity that achieves vascularization and integrates with host tissue.

Various mineral materials like hydroxyapatite (HA), β-tricalcium phosphate (β-TCP), and biphasic calcium phosphate (BCP) have been combined with polymeric matrices to overcome the brittleness of ceramic scaffolds, however, these stable phases exhibit low degradation rates, limiting mineralization and new bone formation.^11^ In contrast to the widely used calcium phosphates, calcium carbonates (CaCO_3_) exhibit rapid dissolution rates especially in vaterite form, which is attributed to its carbonate phase and has been a potential candidate in bone tissue engineering due to its excellent osteoconductivity and biocompatibility.^12^ In addition, vaterite has been shown to be effective for delivering growth factors, drugs, cytokines, and anti-infectives, which provides a promising design of drug-loaded composite scaffolds for bone regeneration.^13^

In this study, we report the use of calcium carbonate (CaCO_3_) particles, in vaterite crystalline form, that have been shown to be highly effective in promoting the biomimetic formation of hydroxyapatite (HA) as opposed to using hydroxyapatite. In addition, vaterite has a porous structure with a large surface area that exhibits excellent biocompatibility, biodegradability, and greater hydrophilicity. The relatively high rate of release of calcium ions from vaterite not only favors transformation into apatite,^13^ but the dissolution kinetics also leads to in situ pore formation that can enhance nutrient transport through the whole thickness of the scaffold. The Fe_3_O_4_ nanoparticles in this study were single domain SPIONs that can generate an intrinsic magnetic field at the nanoscale level. The magnetic composite scaffolds were fabricated using vaterite particles as fillers with a poly(vinyl alcohol) (PVA) matrix, cross-linked by freeze thawing (FT) cycles that obviates the use of any cross-linking agents, while conferring porosity, also enabling fluid uptake and nutrient transport. MNPs were incorporated to generate a magnetic field and improve mechanical and biological properties. Scaffolds with different concentrations of MNPs were fabricated, and the morphology, mechanical and physicochemical properties, mineralization, and in vitro biocompatibility were evaluated.

## 2 Materials and Methods

### 2.1 Materials

Ferric trichloride hexahydrate (FeCl_3_·6H_2_O, AR, 97%), poly(acrylic acid) (PAA, Mw ∼ 2000) and sodium acetate (BioXtra, ≥99.9%), ethylene glycol (EG, AR, ≥99.5%), calcium chloride (CaCl_2_, GR, ≥96%), sodium carbonate (Na_2_CO_3_, AR, ≥99.9%), and casein (BR) were used to fabricate MNPs and vaterite particles. PVA (Mw: 145 000 Da, ≥98% hydrolyzed) was used as the matrix. Hydroxyapatite (nanopowder, <200 nm particle size, ≥97%), sodium fluoride (BioXtra, ≥99%), 69% nitric acid, and bis-Tris (≥98.0%, titration) were used to fabricate the mineralization solution. All the reagents were used as received without any further purification. Deionized water was used to prepare solutions throughout the experiments.

### 2.2 Preparation of Magnetic Composite Scaffolds

MNPs were fabricated using a solvothermal method as described previously.^13^ In brief, ferric chloride hexahydrate (0.54 g), PAA (0.03 g), and sodium acetate (3.5 g) were dissolved and magnetically stirred in 20 mL of ethylene glycol at 55 °C. The yellowish-brown mixture was then poured into a 25 mL Teflon-lined stainless steel autoclave, heated to 200 °C, maintained for 10 h, and then cooled to room temperature. MNPs were subsequently collected via magnetic separation, washed three times with ethanol, and then dried at 60 °C under vacuum.

Vaterite particles were synthesized by a fast coprecipitation method.^14^ Casein (0.4 g) was dissolved in 100 mL of Na_2_CO_3_ solution (0.2 M) and magnetically stirred at 30 °C. The CaCO_3_ precipitates were then obtained by adding 100 mL of CaCl_2_ solution (0.2 M) dropwise into the mixture while stirring over 30 min. The vaterite particles were collected, washed with deionized water, freeze-dried, and stored in an airtight dry container until further use.

For the PVA-Vaterite magnetic composite scaffolds, MNPs (0, 1, 3, and 6% of total weight) were first dispersed homogeneously in the 10% (w/v) PVA solution by ultrasonication. The suspension was then mixed thoroughly with vaterite particles at a ratio of PVA:vaterite = 40:60 by weight according to our previous study,^15^ and cylindrical specimens of 6 mm diameter and 12 mm height were prepared. The specimens were then frozen at −80 °C for 1 h, freeze-dried for 24 h, and thawed for 24 h at room temperature. The condenser temperature (−57.5 °C) and vacuum pressure (125 mTorr) of the freeze-dryer (VirTis BenchTop Pro, SP Scientific) were kept constant. The freeze–thaw cycle was repeated twice to make magnetic composite scaffolds.

### 2.3 Characterization of Scaffolds

The crystalline phases of MNPs and vaterite particles were examined using X-ray powder diffraction (XRD, PANalytical X’Pert Pro, Netherlands) with Cu Kα radiation at 45 kV and 40 mA. Raman spectra (Renishaw inVia, UK) with 785 nm laser, 1200 L/mm grating, and 5% laser power were recorded. The morphology of MNPs was observed by transmission electron microscopy (TEM, JEM-1400Plus, JEOL, Japan), and the size distribution was analyzed by randomly measuring 50 nanoparticles under the microscope. The zeta potential of MNPs was measured by dynamic light scattering (DLS, Zetasizer, Malvern Panalytical, UK). The morphology of the scaffolds was visualized using a scanning electron microscope (SEM, JCM-6000plus, JEOL, Japan) equipped with an energy-dispersive X-ray spectroscope (EDS). The 3D structure of scaffolds was scanned by microcomputed tomography (μCT, μCT50, SCANCO, Switzerland) rotating 360° at a pixel resolution of 6 μm, 45 kV peak voltage, and 200 μA with air filter. The magnetic property measurements were carried out on a vibrating sample magnetometer (VSM, PPMS, Quantum Design, UK) at room temperature.

### 2.4 Compression Test

The static mechanical properties of the scaffolds were determined using a universal testing machine (Instron 5569A) with a 50 kN load and at a crosshead speed of 5 mm/min. The compression test was carried out in accordance with ISO 291 and ISO 604 (compression tests for plastics) using dry and fully hydrated cylindrical specimens (6 mm diameter and 12 mm height; *n* = 6).

### 2.5 Water Uptake Study

The dry weight of the specimens was recorded before hydration in deionized water at 37 °C, and weight changes were monitored over several time points until equilibrium. The equilibrium water content (EWC) was determined by eq 1
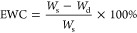
1

The swelling ratio (SR) was calculated as shown in eq 2
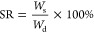
2

*W*_s_ is the weight of the hydrated specimen at equilibrium and *W*_d_ is the initial dry weight of the specimen before hydration.

### 2.6 Degradation Study

Specimens were maintained in deionized water at 37 °C and weighed in the hydrated state at different time points for up to 3 months. The weight change was calculated using eq 3
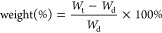
3

The immersion water was refreshed at each time point. The degradation fraction or total loss was calculated using the difference of the initial weight of the dry specimen and the final dry weight of the specimen after hydration, as shown in eq 4
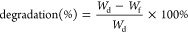
4

*W*_t_ is the weight of the hydrated specimen at each time point, *W*_d_ is the initial dry weight of the specimen before hydration, and *W*_f_ is the final dry weight of the specimen after a 3 month immersion.

### 2.7 Mineralization

The mineralization assay was prepared by mixing hydroxyapatite (0.6027g) and sodium fluoride (0.0504g) in 600 mL of deionized water followed by dropwise addition of 69% nitric acid to completely dissolve the powder. Bis-Tris was used to readjust the pH to 6.00.^16^ The scaffold specimens were placed in 50 mL airtight tubes groupwise and incubated at 37 °C for 7, 14, and 21 days. The mineralized structures were observed and analyzed by SEM and EDS. Raman spectroscopy was used to analyze the crystal forms on the specimens.

### 2.8 In Vitro Biological Evaluation

Preliminary cell viability, proliferation, and adhesion were examined by MTT, alamarBlue, and live/dead staining assays using human osteoblast-like cells (HOS TE85). Cells were preseeded in 96-well plates with a concentration of 1 × 10^4^ cells/well, cultured for 24 h, and then exposed to eluants with different concentrations for 24 and 72 h for the MTT assay. All the eluants were prepared in complete culture medium with a ratio of material to medium = 0.025 g/mL and diluted to 0, 10, 20, 40, 60, 80, and 100% by adding the corresponding volume of medium. Cell proliferation was studied by microseeding cells (2.24 × 10^4^ cells/scaffold) onto the scaffolds (6 mm in diameter) and culturing for up to 28 days in the complete culture medium. The medium was replaced by alamarBlue working solution at different time points, and after culturing for 4 h, the absorbance of the solution was analyzed at 570/620 nm for relative proliferation. Cell adhesion was investigated by collecting cell-seeded specimens at different time points (1, 3, and 7 days) and staining with a viability/cytotoxicity kit (Invitrogen, L-3224). Live cells were stained green by calcein AM (2 μM), indicating intracellular esterase activity, and dead cells were stained red by Ethidium homodimer-1 (EthD-1, 4 μM) binding with nucleic acid due to loss of membrane integrity. Cell morphology was further explored by fluorescence imaging of the cytoskeleton and nucleus. The microseeded scaffolds at each time point (1, 3, and 14 days) were fixed with 4% paraformaldehyde in PBS, permeabilized by 0.1% Triton X-100, and then stained with Alexa Fluor 488 Phalloidin for F-actin and 4,6-diamidino-2-phenylindole (DAPI, Alexa Fluor) for the nucleus. The cells were observed under a fluorescence microscope (Olympus IX51) and imaged via a connected digital camera. The cell morphology of the fixed cell-seeded specimens at time points 1, 14, and 28 days was further examined under a scanning electron microscope (JCM-6000plus, JEOL, Japan).

### 2.9 Statistical Analysis

Data are presented as mean ± standard deviation (SD). Comparative tests (independent *t* tests, one-way ANOVA, and post hoc Tukey analysis) were performed accordingly using GraphPad Prism 9 (*p < 0.05, **p ≤ 0.01, ***p ≤ 0.001, and ****p ≤ 0.0001).

## 3 Results and Discussion

### 3.1 Characterization of MNPs and Magnetic Scaffolds

**Figure 1:**
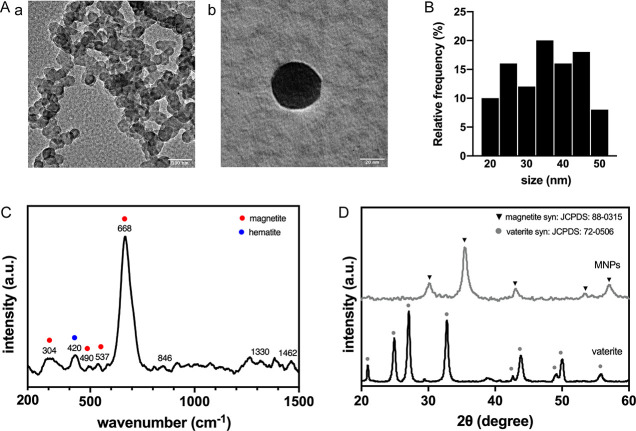
(A) TEM images of (a) homogeneous spherical MNPs and (b) single magnetic nanoparticle. (B) Particle size and size distribution of MNPs. (C) Raman spectrum of MNPs. (D) XRD spectra of MNPs and vaterite particles showing a typical magnetite crystalline phase of MNPs and vaterite phase of CaCO_3_ particles.

The XRD diffraction pattern of vaterite particles (Figure 1D) revealed the typical vaterite phase (JCPDS #72-0506). The diffraction peaks of the synthesized MNPs at 30.1, 35.5, 43.1, 53.5, and 57.0° assigned to (220), (311), (400), (422), and (511) planes demonstrated a typical magnetite crystalline phase of MNPs (JCPDS #88-0315). The crystal size calculated by Scherrer formula based on the most intense peak (311) was 21.81 nm, indicating a single domain superparamagnetic magnetite crystal.^17^ The zeta potential of MNPs was −46.37 ± 4.26 mV, which demonstrated the negative charge of MNPs and their great stability in suspension.

The TEM images in Figure 1A showed the formation of uniform spherical MNPs that were prepared using a solvothermal method. The particle size and size distribution of the magnetic nanoparticles ranged between 20 to 50 nm with an average of 35.15 nm (Figure 1B), and the size difference is due to the slight agglomeration of nanocrystals within the dispersion. The Raman spectrum of MNPs showed characteristic vibration bands of magnetite with peaks at 304 (E_g_), 450–490 (T_2g_), 537 (T_2g_), and 668 cm^–1^ (A_1g_) (Figure 1C).^18^ The peak at 420 cm^–1^ (E_g_) corresponds to hematite that indicated a moderate crystalline transition on the surface under the laser power.^19^

**Figure 2:**
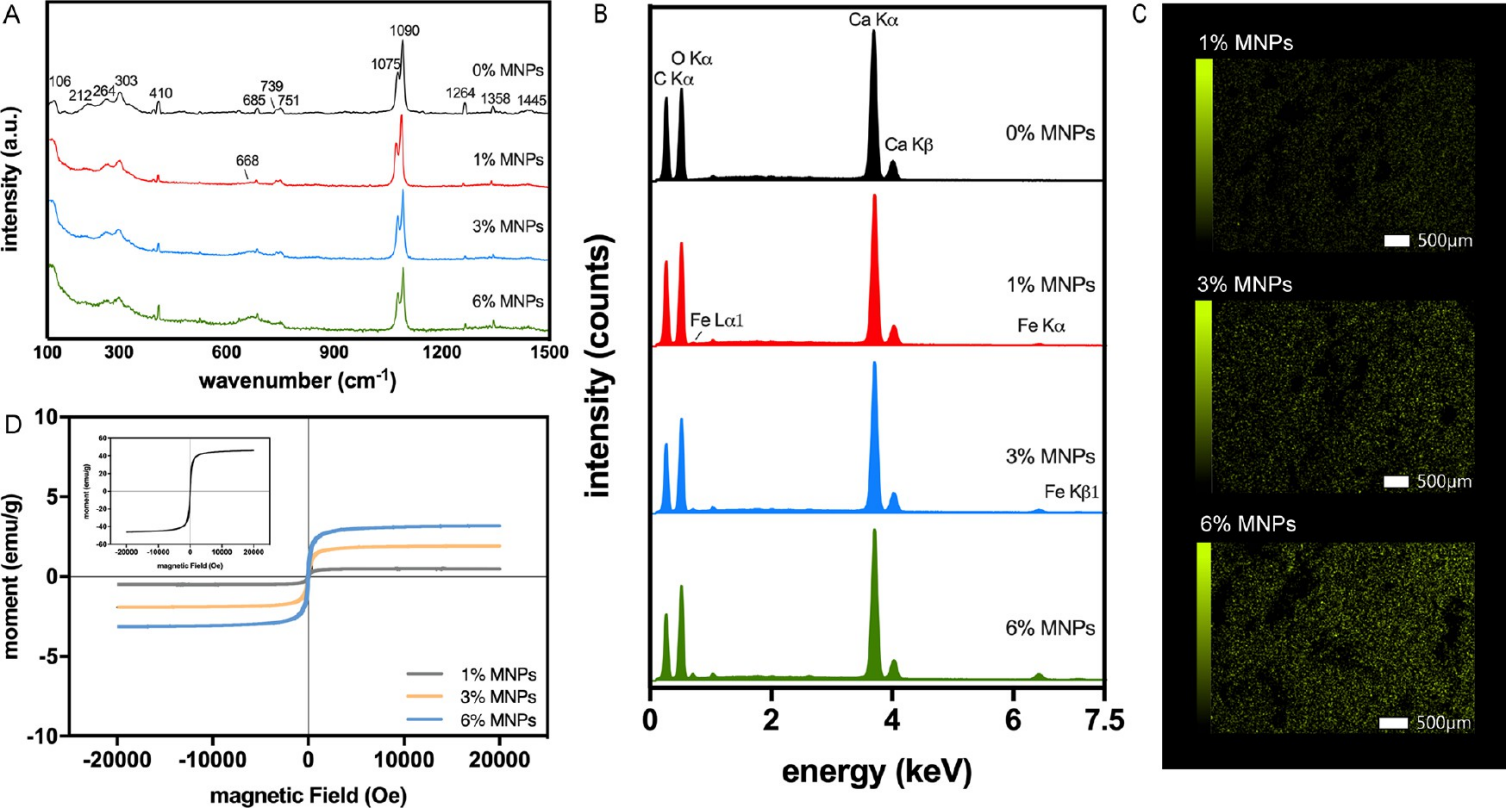
(A) Raman spectra of PVA-Vaterite composite scaffolds with 0, 1, 3, and 6% MNPs showing characteristic peaks of vaterite particles and increased peak intensity of MNPs with higher concentration. (B) EDS spectra of PVA-Vaterite composite scaffolds with 0, 1, 3, and 6% MNPs and (C) EDS mappings of Fe K showing a uniform distribution of MNPs within the scaffolds. (D) Magnetization loops of PVA-Vaterite magnetic scaffolds with 1, 3, and 6% MNPs and (inset) MNPs.

The Raman spectra of PVA-Vaterite scaffolds with 0, 1, 3 and 6% of MNPs (Figure 2A) showed characteristic peaks of vaterite particles including 106, 212, 264, and 303 cm^–1^ (lattice mode), 685, 739, and 751 cm^–1^ of *v*_4_ (in-plane bending mode), and 1075 and 1090 cm^–1^ of ν_1_ (symmetric stretching mode).^20^ The peaks at 410 and 1264 cm^–1^ were assigned to casein within the vaterite particles, and peaks at 1358 and 1445 cm^–1^ correspond to the CH bending and CH_2_ bending/scissors of PVA.^21,22^ The characteristic peak at 668 cm^–1^ due to magnetite was observed to increase with increasing concentration of MNPs, confirming the incorporation of MNPs within the scaffolds, which also demonstrated that the stable magnetite phase of MNPs was not affected by the FT cycles. The spectral data confirmed that the composite scaffolds were successfully formed by combining vaterite particles as filler with cross-linked PVA matrix.

The EDS maps (Figure 2C) showed a uniform distribution of the Fe element on the scaffold surface, indicating homogeneous distribution of MNPs, which can be attributed to the dispersion of MNPs in PVA as it can act as a stabilizer limiting agglomeration of the nanoparticles.^23^ The spectra (Figure 2B) revealed an increasing intensity of the peak, Fe Kα, with an increasing concentration of MNPs in the scaffolds. The quantitative results of EDS showed that the mass % of Fe in groups 1% MNPs, 3% MNPs, and 6% MNPs were 0.82 ± 0.14%, 2.42 ± 0.09%, and 4.46 ± 1.12%, respectively, and the mass % of MNPs were calculated to be 1.14 ± 0.19%, 3.36 ± 0.13%, and 6.19 ± 1.56%, which are consistent with the amount of MNPs included during the fabrication, indicating that almost all the MNPs were well incorporated within the scaffolds.

The magnetic properties of MNPs and the PVA-Vaterite scaffolds with MNPs were evaluated by VSM at room temperature. The MNPs exhibited a superparamagnetic behavior with magnetization saturation (*M*_s_) of 56.31 emu/g, which is in agreement with the result calculated from the XRD spectrum showing that the MNPs were superparamagnetic single domain nanoparticles with primary nanocrystal sizes below 30 nm.^17^ MNPs in colloid solution are better at displaying superparamagnetic behavior due to the magnetic relaxation of the chain-like structures formed in solution and the metastable Brownian dynamics of the nanoparticles, which can improve the magnetic property of the PVA-Vaterite scaffolds with MNPs homogeneously dispersed in the PVA solution.^17,23^ The superparamagnetic behavior was observed in all of the MNP-containing scaffolds (Figure 2D). The magnetization saturation values of magnetic scaffolds with 1, 3, and 6% MNPs were 0.48, 1.93, and 3.16 emu/g, respectively. The values of *M*_s_ increased with the increase in MNPs and were proportional to the concentration of MNPs incorporated in the scaffolds during fabrication, which is important in the adjustment of magnetic intensity of scaffolds.

**Figure 3:**
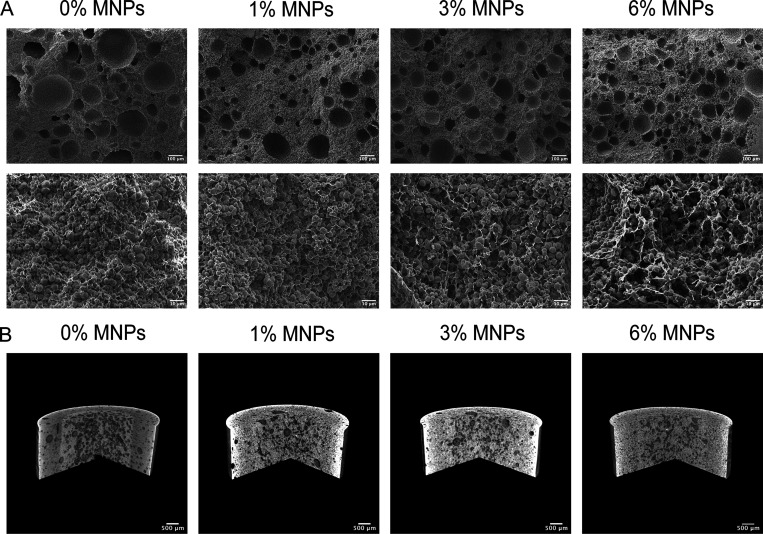
(A) SEM images of PVA-Vaterite composite scaffolds with 0, 1, 3, and 6% MNPs at magnifications of 100× and 1000× showing a cross-linked porous structure with densely packed vaterite particles. (B) μCT images exhibiting the 3D porous structure of composite scaffolds with a slightly decreased porosity of magnetic scaffolds.

The SEM images (Figure 3A) of PVA-Vaterite scaffolds exhibited a porous structure with decreasing pore size with increasing concentration of MNPs. At a higher magnification, the homogeneous distribution of vaterite particles in the scaffolds was apparent, which is vital to yield optimum mechanical properties.^24^ The μCT images (Figure 3B) also evidenced the 3D porous structure of all of the composite scaffolds with a slightly denser structure and lower porosity of magnetic scaffolds (Table 1). However, no significant difference was observed among magnetic scaffolds with different concentrations of MNPs. The open and interconnected porous structure of PVA-Vaterite scaffolds resulted from freezing–thawing of PVA to form the cross-linked network. The large-scale pores were likely formed via the freezing–thawing process due to the ice crystal formation and sublimation, while the small-sized pores between cross-linked PVA chains resulted from the intermolecular interactions between the pendant hydroxyl groups of PVA,^25^ which are critical for cell infiltration, osteogenesis, nutrient transport, and tissue in-growth. The uniform spherical shape of vaterite particles was not affected by the FT cycles nor the incorporation of MNPs. However, with a higher concentration of MNPs, the filler particles tended to be less densely embedded in the matrix, leaving more small-sized pores among the interconnecting cross-linked PVA chains. Additionally, vaterite particles acted partially as a porogen during the fabrication due to its high dissolution rate.^26^ With the incorporation of MNPs, the hydroxyl groups of PVA chains can undergo complexation with ferric ions, resulting in a strong hydrogen bonding between MNPs and PVA chains, which enhanced the cross-linking, thereby limiting PVA chain mobility. This can be attributed to the smaller ice nuclei formation during the freezing–thawing, causing a slightly lower porosity and smaller pore sizes.^23^ On the other hand, the presence of MNPs can interfere with the weak electrostatic interaction between vaterite (Ca^2+^) and PVA (−OH^–^),^27^ exposing more vaterite particles to the aqueous solution, leading to a relatively accelerated dissolution and increased microporosity among cross-linked PVA chains, therefore providing a sufficient surface area and pore volume for cell anchorage and nutrient transport without interfering with the mechanical support.

**Table 1:** Total Volume, Pore Volume, and Total Porosity of PVA-Vaterite Composite Scaffolds with Increasing Concentration of MNPs^a^

composite scaffolds	total volume (mm^3^)	pore volume (mm^3^)	total porosity (%)
0% MNPs	109.92 ± 2.65	19.72 ± 1.32	17.94 ± 1.20
1% MNPs	117.31 ± 3.43	18.45 ± 0.88	15.72 ± 0.75*
3% MNPs	111.55 ± 3.24	16.78 ± 0.48	15.04 ± 0.42*
6% MNPs	111.20 ± 0.57	16.91 ± 0.85	15.21 ± 0.77*

a Data are presented as mean ± SD, *n* = 3 and compared to the values of 0% MNPs. *p < 0.05, **p ≤ 0.01, ***p ≤ 0.001, and ****p ≤ 0.0001. No significant difference among 1, 3, and 6% MNPs was observed.

**Figure 4:**
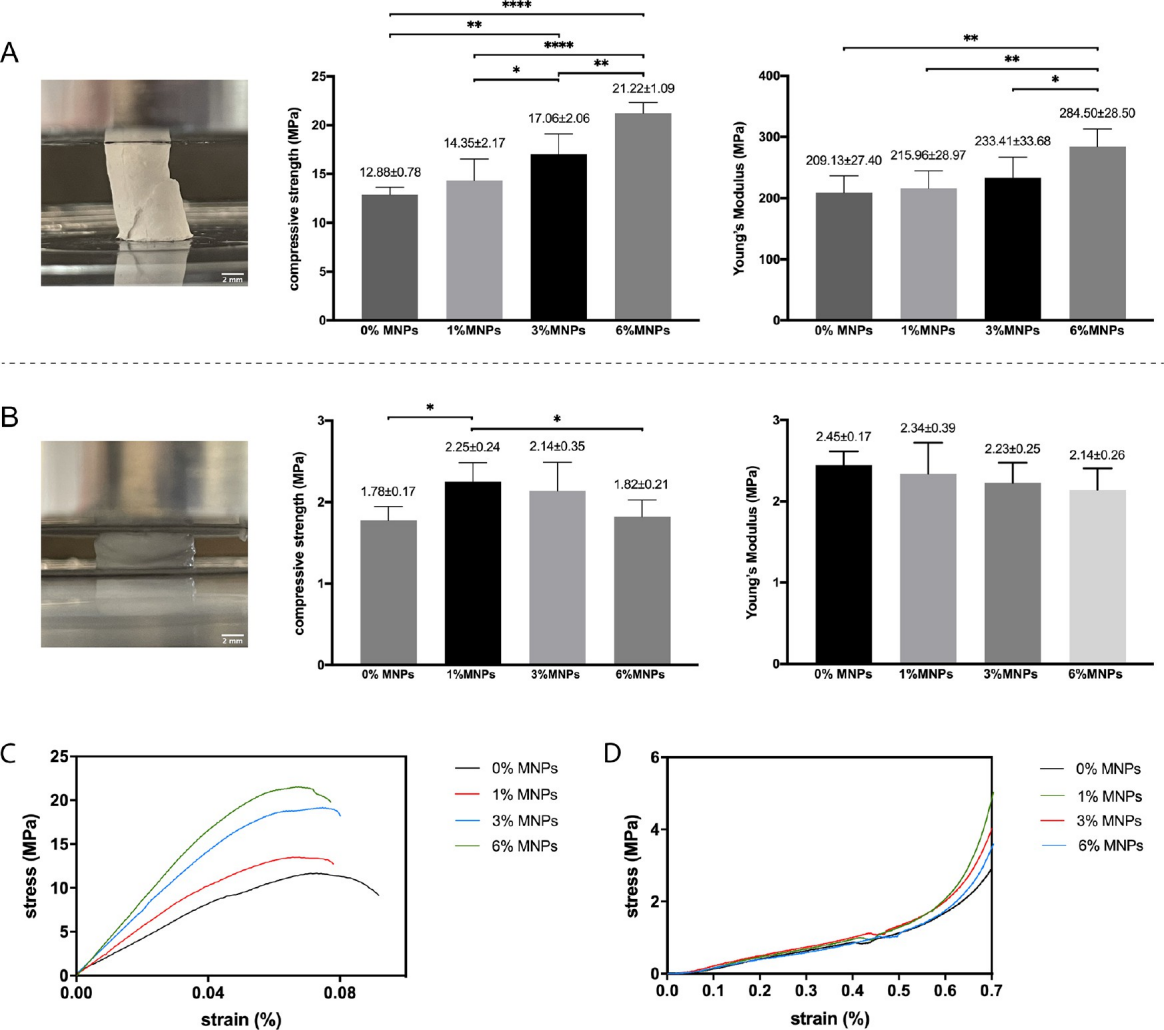
(A, B) Representative images of PVA-Vaterite scaffolds tested in compression, compressive strength, and Young’s modulus of composite scaffolds in (A) dry and (B) hydrated states. Data are presented as mean ± SD, *n* = 6, *p < 0.05, **p ≤ 0.01, ***p ≤ 0.001, and ****p ≤ 0.0001. (C, D) Representative stress–strain curves of the scaffolds tested in dry (C) and hydrated (D) states.

The densely packed filler particles and MNPs in the scaffolds improved the mechanical properties of composite scaffolds with the dry scaffolds exhibiting brittle fracture while the fully hydrated scaffolds being compressible. As shown in Figure 4A, the dry scaffolds were loaded in compression to fracture with typical diagonal splitting patterns. Meanwhile, the hydrated scaffolds did not undergo brittle fracture until maximum compressibility (Figure 4B), and thus, the values of compressive strength were taken at maximum compressibility and Young’s modulus was calculated at a low strain (10%) to capture the initial linear elastic region (Hooke’s law).^28^ In the dry state, the magnetic scaffolds exhibited a statistically significant increase in both compressive strength and Young’s modulus with increasing concentration of MNPs, indicating an improved strength and stiffness that could be attributed to the strong interactions between MNPs and PVA through hydrogen bonding and physical cross-linking.^23^ In contrast, the hydrated magnetic scaffolds showed a reverse trend with a decrease in compressive strength with 6% MNPs (Figure 4B), which was probably due to the rapid dissolution of vaterite particles that were separated and exposed by MNPs. However, the compressive strength values of all hydrated magnetic scaffolds were higher than the value of non-magnetic scaffold, and no significant difference was observed in Young’s modulus, which demonstrated the improved mechanical properties of magnetic scaffolds in both dry and hydrated states.

The porous structure and formation of PVA network resulted in EWCs ranging between 32.32 to 42.8% in deionized water at 37 °C (Table 2) and is likely to enable fluid transport, penetration of oxygen, and various nutrients for osteogenesis and angiogenesis necessary for bone regeneration. The composite scaffolds with MNPs exhibited a statistically significantly lower EWC compared to non-magnetic scaffolds, which corresponds to the relatively lower porosity. This can be attributed to the interaction of PVA and MNPs, which limits the mobility of PVA chains, lowering the volume of amorphous regions in the matrix, leading to a higher cross-linking density.^29^ All the scaffolds exhibited an appropriate and controlled swelling capability, which is beneficial for balancing adequate mechanical properties and biological fluid absorption, and this can assist in overcoming necrosis of the surrounding tissue that is usually observed due to the excessive swelling of scaffolds, causing pressure damage.^30^

**Table 2:** Equilibrium Water Content (EWC) and Swelling Ratio (SR) of PVA-Vaterite Composite Scaffolds with Increasing Concentration of MNPs^a^

composite scaffolds	EWC (%)	SR
0% MNPs	42.80 ± 1.34	1.75 ± 0.04
1% MNPs	32.67 ± 0.61****	1.49 ± 0.01****
3% MNPs	32.32 ± 1.38****	1.48 ± 0.03****
6% MNPs	34.92 ± 0.58****	1.54 ± 0.01****

a Data are presented as mean ± SD, *n* = 3 and compared to the values of 0% MNPs. (*p < 0.05, **p ≤ 0.01, ***p ≤ 0.001, and ****p ≤ 0.0001). No significant difference among 1, 3, and 6% MNPs was observed.

**Figure 5:**
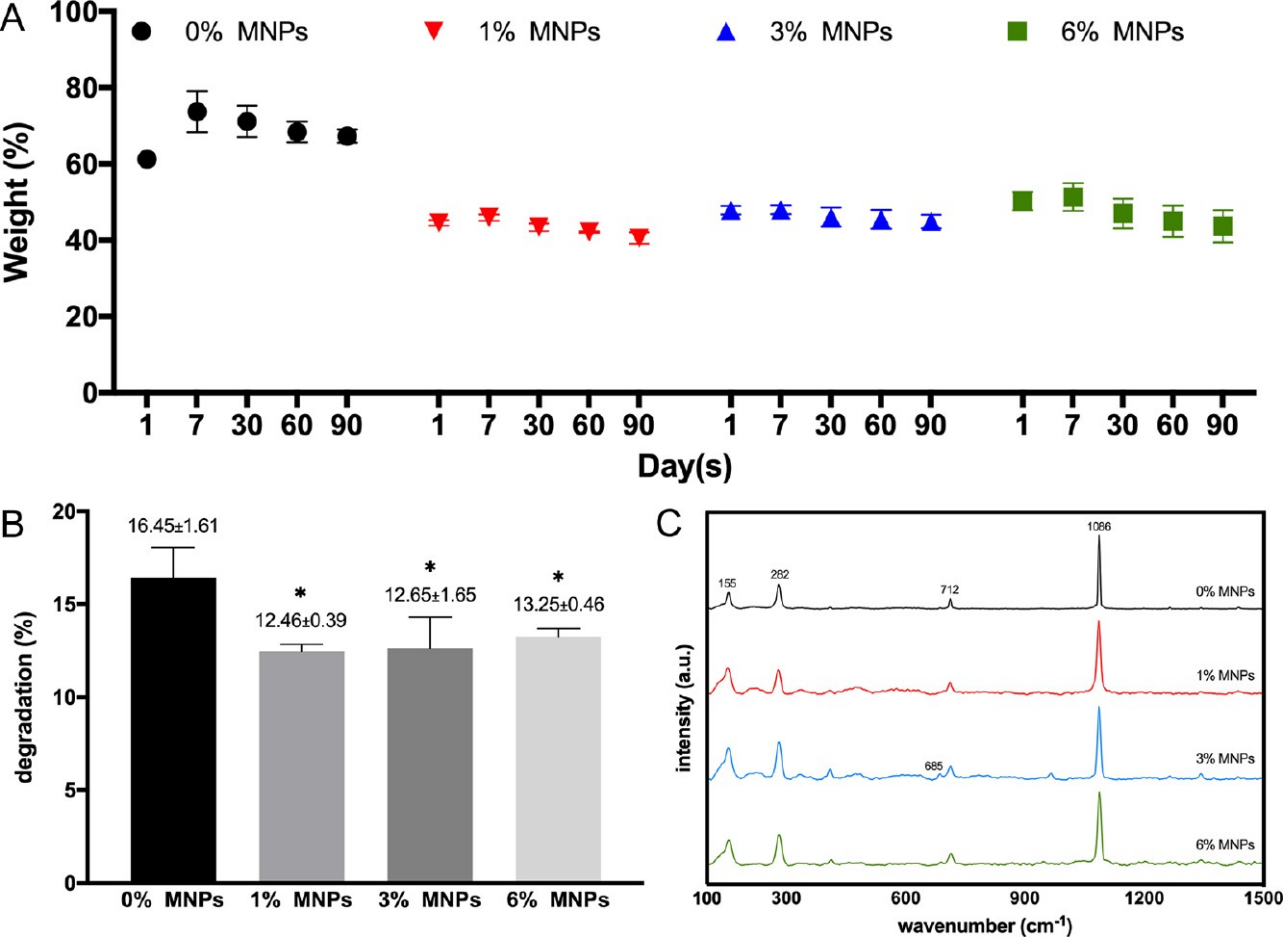
(A) Gravimetric changes over time in deionized water in percentage, showing slower degradation and higher structural stability of magnetic scaffolds. (B) Degradation fraction or total loss (%) of composite scaffolds immersed in deionized water for 3 months. (C) Raman spectra of composite scaffolds immersed in deionized water for 3 months exhibiting peaks of calcite phase, demonstrating the dissolution and recrystallization of vaterite particles in water.

The hydrolytic degradation over 3 months is a complex process since water uptake and degradation occur simultaneously due to absorption of water by the hydrogel matrix and loss of vaterite particles.^31^ This is reflected in the initial increase in the weight (Figure 5A) followed by a steady loss. The magnetic scaffolds exhibited a lower EWC and lower degradation fraction (Figure 5B) since incorporation of MNPs increased the structural integrity due to the intermolecular forces in play within the PVA matrix. This rendered the scaffolds less susceptible to hydrolysis and degradation within a reasonable time to balance between degradation and sufficient mechanical support.^15,23^ Due to the higher solubility, vaterite is reported to transition into calcite in aqueous phases,^32^ which was confirmed by the Raman spectra shown in Figure 5C. Characteristic peaks at 155 and 282 cm^–1^ (lattice modes), 1086 cm^–1^ (ν_1_ symmetric stretching), and 712 cm^–1^ (ν_4_ in-plane bending) are assigned to calcite, demonstrating the dissolution and recrystallization of vaterite particles during the immersion.^20^ Noticeably, a less intense peak at 685 cm^–1^ corresponding to vaterite was still visible in the spectra of magnetic scaffolds with 3 and 6% MNPs, which also indicated a higher structural integrity and stability with a higher concentration of MNPs.

**Figure 6:**
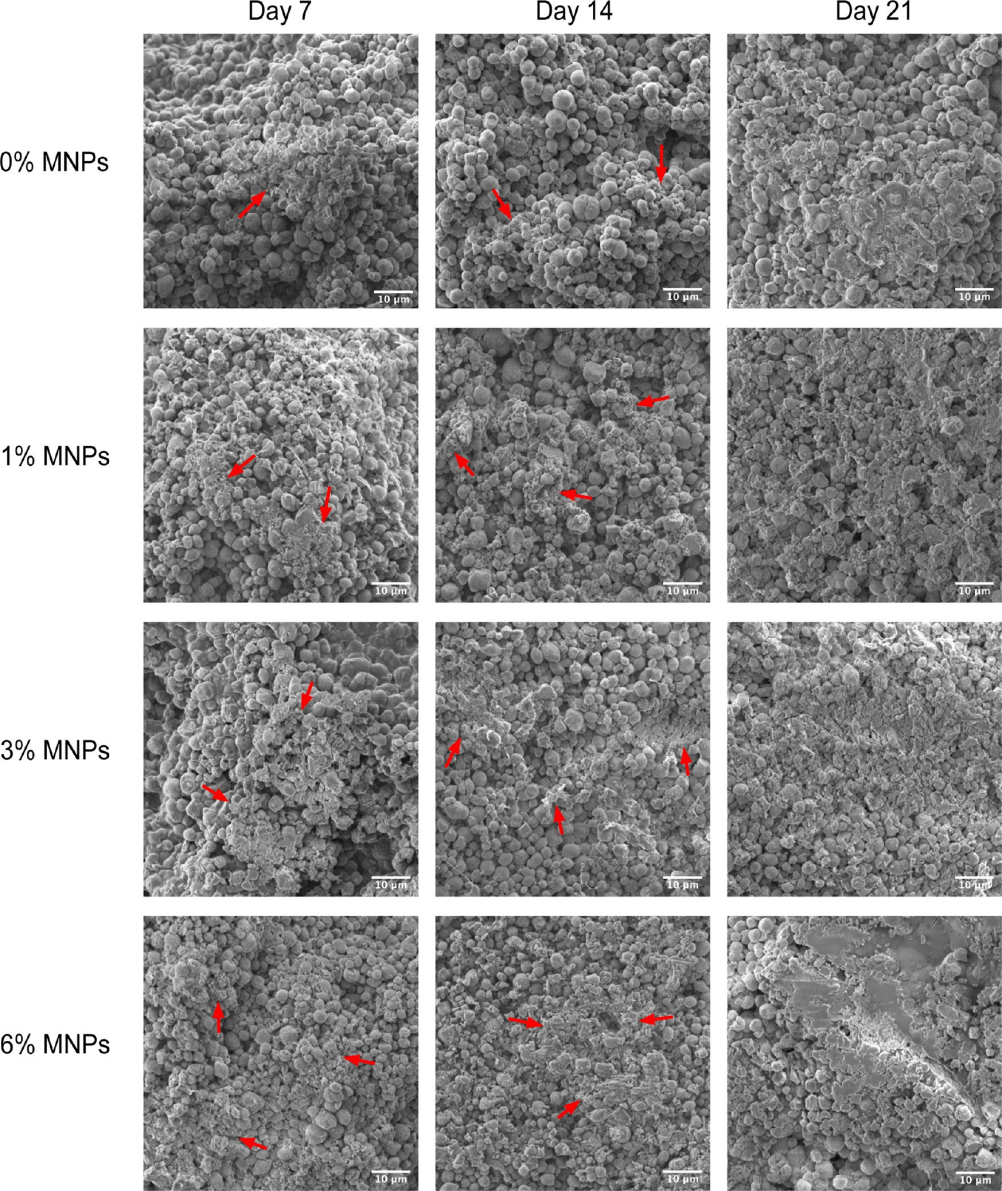
SEM images of composite scaffolds after mineralization for 7, 14, and 21 days. Cauliflower-like apatite crystals were observed on the scaffolds (indicated by red arrows).

**Figure 7:**
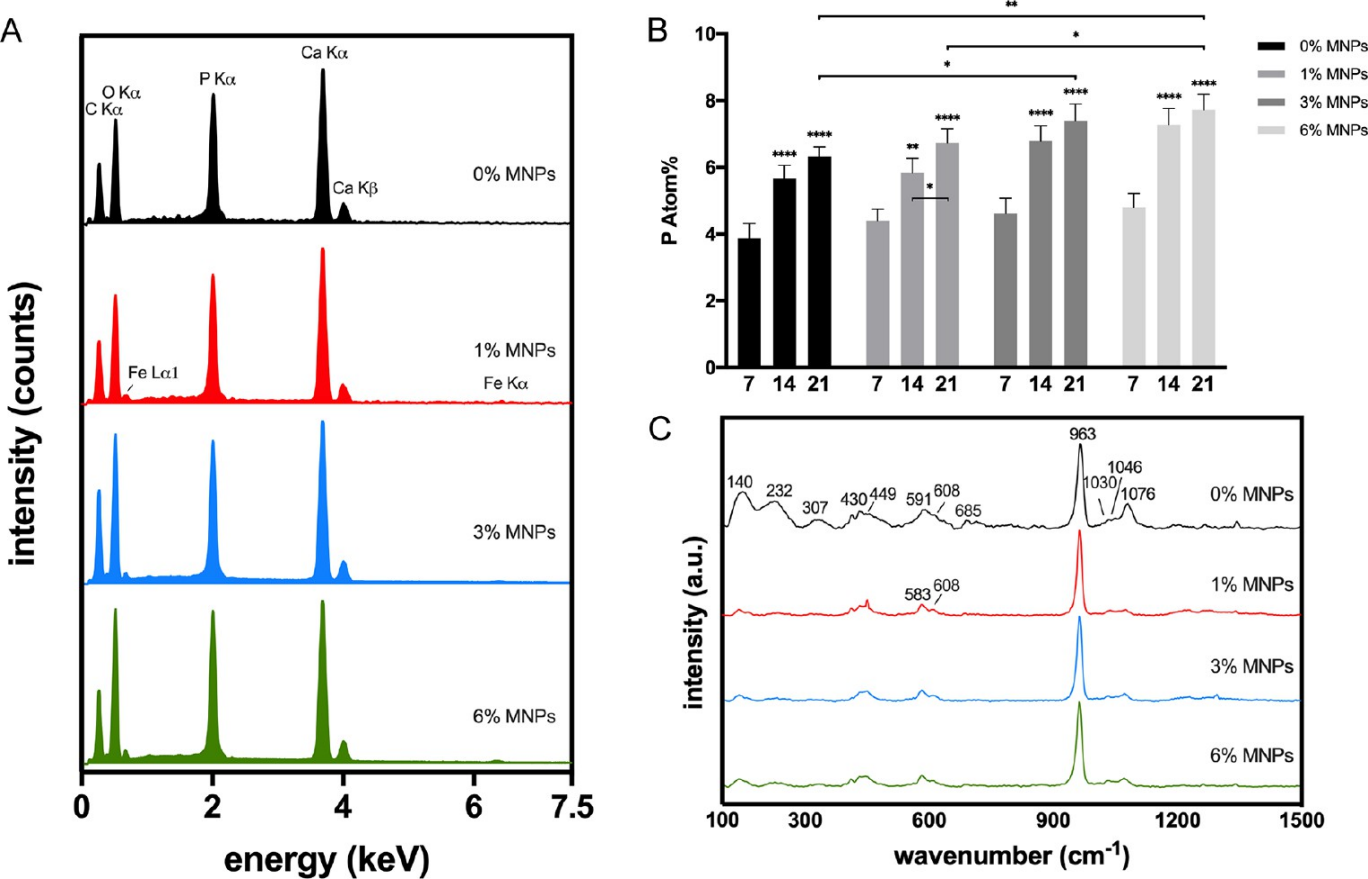
(A) EDS spectra of composite scaffolds after mineralization for 21 days and (B) P atom % from EDS mappings of composite scaffolds after mineralization for 7, 14, and 21 days, showing a significant increase in P atom % on the scaffolds with higher concentration of MNPs (data are presented as mean ± SD, *n* = 3, *p < 0.05, **p ≤ 0.01, ***p ≤ 0.001, and ****p ≤ 0.0001). (C) Raman spectra of composite scaffolds after mineralization for 21 days, with a characteristic peak at 963 cm^–1^ corresponding to apatite.

Scaffolds for bone tissue engineering should enable biomineralization and integration with the surrounding bone.^33^ As shown in Figure 6, the scaffolds in the mineralization solution^16^ exhibited a gradual dissolution of spherical vaterite particles and the deposition of smaller cauliflower-like apatite crystals. The pH was monitored over the immersion period and remained between 7.8 to 8.0, indicating physiological conditions, which would be beneficial to cell growth and biomineralization. With increasing time in the immersion medium, the crystals formed on the scaffolds were observed to spread and connect, overlapping the residual vaterite particles and tending to form intact layers on the matrix. The continuous precipitation of apatite crystals on the surface of the scaffolds is likely to have occurred due to the release of Ca^2+^ ions from vaterite, which attracted PO_4_^3–^ and OH^–^ anions, and then Ca^2+^ cations from solution were attracted by the former anions, thus inducing a continuous formation of apatite crystals on the scaffolds.^34^ The porous structure of composite scaffolds also facilitated ionic diffusion throughout the structure, enabling access to the ionic species, and provided more open space for the crystals to spread until a mineralized layer was formed.^35^ The Ca/P ratio was analyzed by EDS on three random cauliflower-like crystals on the scaffolds, and the values were all close to 1.67 (Table 3), indicating the apatite phase of nucleated crystals. This was further confirmed by Raman spectra, as shown in Figure 7C. The less intense peaks of vaterite compared to the Raman spectra of scaffolds in Figure 2A indicated the gradual dissolution of vaterite. The apatite phase of crystals grown on the scaffolds can be elucidated by the characteristic peaks at 963 cm^–1^ of PO_4_^3–^ ν_1_ (symmetric stretching mode), 430 and 449 cm^–1^ of PO_4_^3–^ ν_2_ (doubly degenerate bending mode of the O–P–O bond), and 1030, 1046, and 1076 cm^–1^ of PO_4_^3–^ ν_3_ (triply degenerate asymmetric stretching mode of the P–O bond), which can be distinguished from other mineral deposits such as amorphous calcium phosphate (950 cm^–1^), monetite (995 cm^–1^), and brushite (985 cm^–1^).^36^ The peaks ranging from 568 to 617 cm^–1^ are assigned to PO_4_^3–^ ν_4_ (doubly degenerate bending mode of the O–P–O bond), and the peaks between 100 and 320 cm^–1^ are lattice modes of HA.^36^

**Table 3:** Atomic Percentage of Ca and P and Ca/P Ratio of Crystals Grown on the Composite Scaffolds after Mineralization for 21 Days^a^

composite scaffolds	Ca	P	Ca/P
0% MNPs	22.54 ± 3.24	13.41 ± 1.60	1.68 ± 0.06
1% MNPs	22.67 ± 1.53	13.69 ± 1.58	1.66 ± 0.10
3% MNPs	22.09 ± 1.56	13.25 ± 0.61	1.67 ± 0.09
6% MNPs	23.39 ± 1.86	13.89 ± 1.05	1.68 ± 0.03

a Data are presented as mean ± SD, *n* = 3 points on the representative scaffolds.

The deposition of apatite crystals was enhanced in the magnetic scaffolds and formed in the early stages of the immersion period (Figure 7A), also supported by the analysis of the atomic percentage of phosphorus from EDS quantitative results (Figure 7B). All scaffolds showed increased apatite formation during the immersion, while a significant increase in P atom (%) on the scaffolds with 3% and 6% MNPs from day 7 to 14 was observed, with a statistically significantly higher P atom (%) on day 21. The EDS spectra of composite scaffolds after mineralization for 21 days also revealed a more intense peak for the P atom on scaffolds with a higher concentration of MNPs (Figure 7A), which indicated an improved apatite formation ability of scaffolds with the incorporation of MNPs. The presence of MNPs increased the overall negative charge on the surface, thus, with increasing concentration of MNPs in the scaffolds, there is a stronger induction capability for apatite formation by attracting Ca^2+^, and MNPs act as nucleating sites for the cation and anion interaction to form apatite nuclei, thus accelerating biomineralization.^37^ These results are supported by similar findings by Radha et al. wherein Fe-nHAp biomaterials with lower degrees of crystallinity and decomposition rates showed a higher ionic release in acellular biomineralization immersion solutions.^33^

### 3.2 In Vitro Biological Evaluation

**Figure 8:**
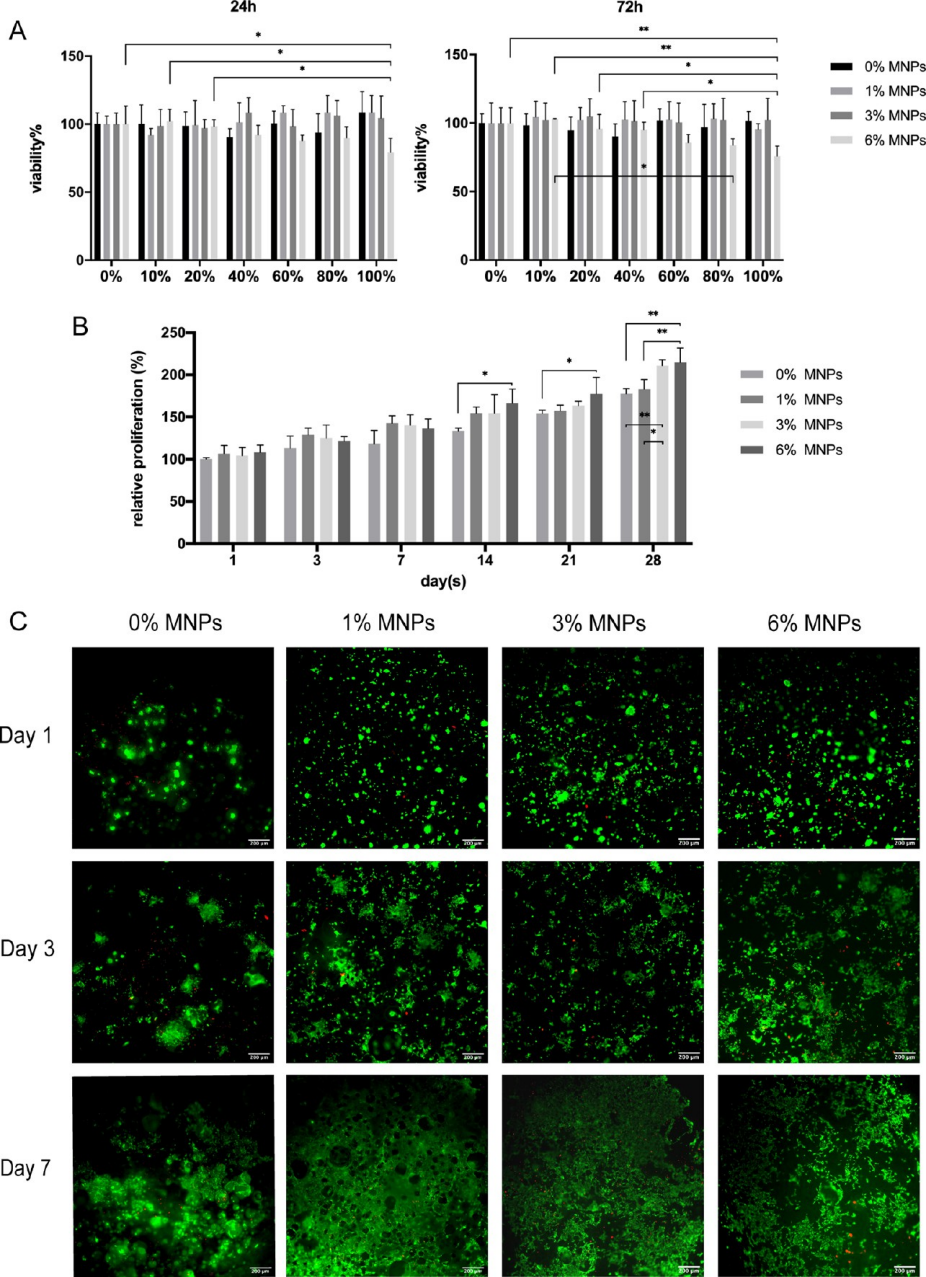
(A) MTT assay showing HOS cell viability after 24 and 72 h exposure to scaffold eluants. (B) alamarBlue assay analyzing the relative proliferation of cells after direct seeding on the scaffolds for up to 28 days. A significant increase in proliferation was observed on scaffolds with increasing concentration of MNPs (*p < 0.05, **p ≤ 0.01, ***p ≤ 0.001, and ****p ≤ 0.0001). (C) Live/dead staining of HOS cells cultured on composite scaffolds after 1, 3, and 7 days. Dense cell sheets were observed on magnetic scaffolds after 7 days.

The eluants from the hydrogel composite scaffolds were exposed to human osteoblast-like cells (HOS) to ascertain any cytotoxicity in the leachants. The results (Figure 8A) showed that the cell viability was above 75% for all scaffolds, however, a slight dose-dependent impact was observed with the eluant of scaffolds with 6% MNPs. The high dose impact of MNPs has previously been reported to be mainly due to the internalization of the iron particles within the cells. This may be connected to ROS-mediated toxicity induced by an iron overload,^38^ or nanoparticles interfering with intracellular signaling pathways,^39^ or due to the alteration of cell morphology leading to a higher nuclei to cytoplasm ratio.^40^ However, this also depends on the characteristics of MNPs, cell features, and culture conditions. The experimental conditions may exaggerate the adverse effect of MNPs in a limiting culture environment, such as static cell cultures, which would be reduced by fluid circulation and regular clearance in vivo. The alamarBlue assay assesses cell viability by directly seeding cells on scaffolds (Figure 8B), and the results showed no significant differences between all groups on days 1, 3, and 7, however, with prolonged culture time at days 14, 21, and 28, higher proliferation rates were shown on scaffolds with higher concentration of MNPs. This suggests a greater affinity of HOS cells to the magnetic scaffolds, which along with regular culture medium changes, eliminated the potential cytotoxic effects of any MNPs that would be leaching out of the scaffolds. The live/dead staining of HOS cells cultured on all scaffolds for up to 7 days showed cells spreading along the porous structures and interconnecting with each other (Figure 8C), indicating an enhanced cell–material interaction on the scaffolds. Compared to nonmagnetic scaffolds, magnetic scaffolds displayed greater cell attachment at day 1 with increased cellular proliferation over the entire surface after 3 days and dense cell sheets were formed after 7 days. The roughened surface topography of magnetic scaffolds may partly be responsible for this as well as the presence of MNPs and the magnetic field generated by MNPs.^41^ The incorporation of MNPs enhanced the cross-linking of matrix, which thus may have provided a more textured and porous structure for cell adhesion and higher surface area for further proliferation. Furthermore, MNPs have also been reported to have a higher affinity to vitronectin, a vital protein for osteoblast adhesion, which can subsequently trigger the integrin-mediated focal adhesion and further promote cell proliferation.^42^

**Figure 9:**
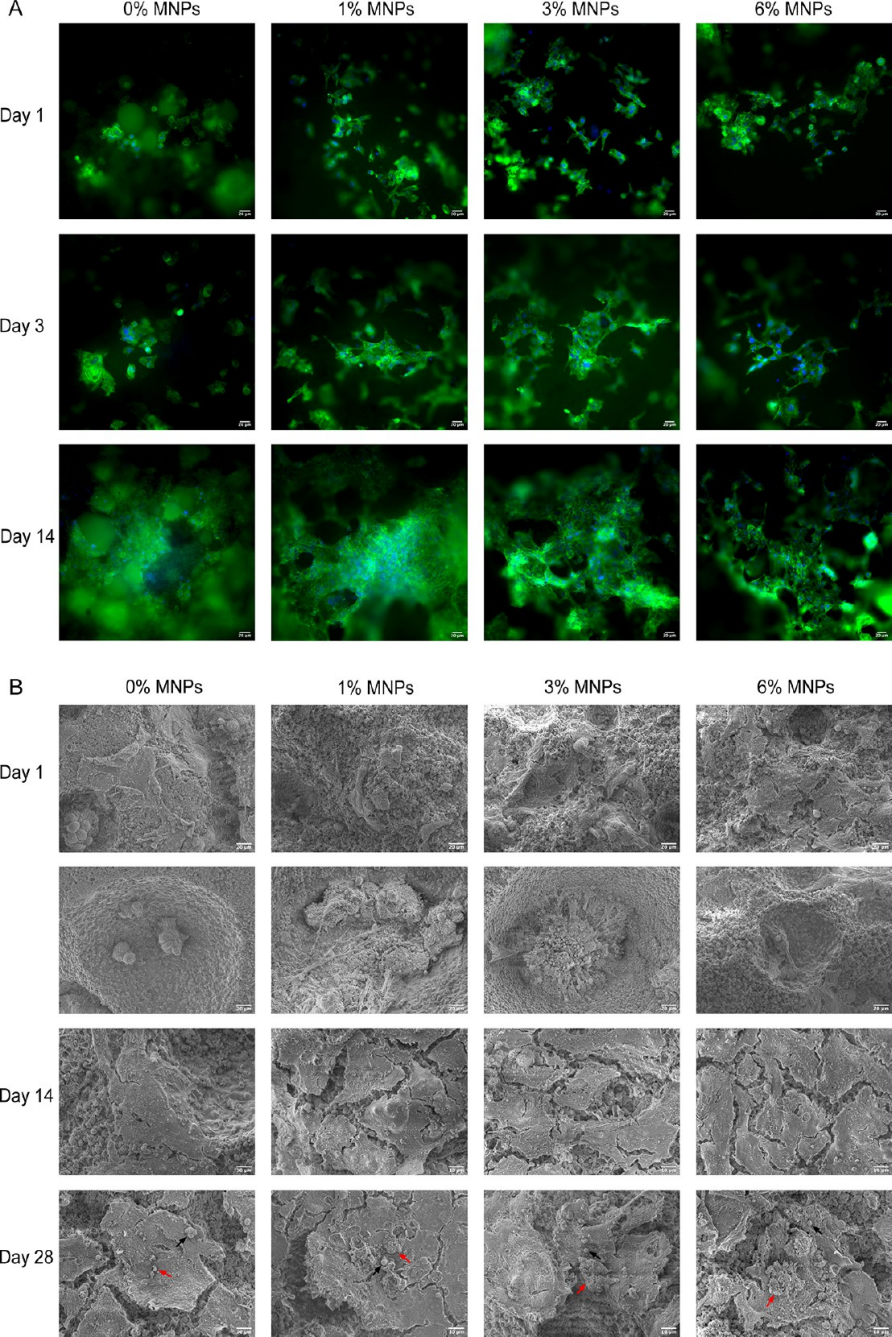
(A) Actin staining of HOS cells cultured on composite scaffolds after 1, 3, and 14 days. The cell skeleton was stained green by phalloidin, and the cell nuclei was stained blue by DAPI. (B) SEM images of HOS cells cultured on composite scaffolds after 1, 14, and 28 days. Cells were attached well both on the surface and within the pores at day 1, with more filopodia observed on cells attached to the magnetic scaffolds. Vaterite particles (indicated as black arrows) and mineral-like nodules (indicated as red arrows) were observed on the cell surface after culture for 28 days.

Cell morphology as observed through actin staining (Figure 9A) showed cell attachment on the magnetic scaffolds with a more flattened cell morphology after day 1, while cells were more rounded and remained as clumps on the nonmagnetic scaffolds. The same phenomenon was observed at day 3, with larger cytoplasmatic protrusions and more cell spreading on the magnetic scaffolds, indicating an enhanced cell–material interaction on the magnetic scaffolds in the early stages. However, by day 14, a three-dimensional cell network growing along the scaffold structure was formed on all composite scaffolds. The cells penetrated and migrated into the internal structures through interconnected pores, demonstrating a great cell affinity to both magnetic and non-magnetic scaffolds. No difference in the cell density on the surface of scaffolds was observed with increasing concentration of MNPs, however, as shown from the alamarBlue data (Figure 8B), there was an increase in cellular activity for the higher MNP-containing scaffolds. This can be attributed to the cells invading into the pores within the scaffolds. Similarly, the SEM images (Figure 9B) showed more filopodia of HOS cells on magnetic scaffolds after 1 day in culture and higher cellular processes and roughness on the surface of cell layers after 14 days, suggesting greater activity of cells on the magnetic scaffolds. At 28 days, cell sheets covered the surfaces of all the scaffolds with numerous cell filopodia and a roughened morphology with mineral-like nodules. Previous studies have shown that the increased deposition of mineral-like nodules on cells cultured in the presence of magnetic scaffolds is due to the stimulatory effect of MNPs on the integrin-mediated, MAPK/NF-κB-activated signaling pathways and BMP-2, p-Smad1/5/8-activated pathways, which are able to induce osteogenesis-related gene expression such as Runx2, thus promoting osteoblast differentiation and bone biomineralization.^43^ Further studies are required to investigate the effect of MNPs on the expression of key osteogenic and angiogenic proteins.

## 4 Conclusions

Porous magnetic calcium carbonate-poly(vinyl alcohol) hydrogel composites were successfully developed by using a freeze–thawing process with magnetite nanoparticles. The magnetite nanoparticles were synthesized using a solvothermal process, which exhibited superparamagnetic properties, and the predispersion of these particles in PVA for fabrication of the composites enabled a homogeneous distribution and limited agglomeration of MNPs in the composites. The incorporation of MNPs significantly improved the mechanical properties and structural integrity of composite scaffolds via strong hydrogen bonding and cross-linking with the PVA matrix. In addition, the degradation data confirmed the gradual loss of vaterite particles with favorable swelling dynamics to facilitate biological fluid absorption. The magnetic scaffolds also facilitated apatite crystal formation due to the gradual dissolution of vaterite particles and the induction capability of MNPs, which also improved cell adhesion and proliferation. However, additional research and experimentation are imperative, specifically to assess the osteogenic and angiogenic potential of these scaffolds.

## Data Availability

The raw/processed data required to reproduce these findings cannot be shared at this time as the data also forms part of an ongoing study.

## Supporting Information Available

pH changes during the mineralization study, SEM images of interconnected macropores within the scaffolds, cross-sectional SEM images of mineralized scaffolds, and depth of cell infiltration in scaffolds (PDF)
