# Supplementary material for: Hydrogel Composite Magnetic Scaffolds: Toward Cell-Free In Situ Bone Tissue Engineering
Source: ACS Appl Bio Mater. 2023 Dec 18;7(1):168–81. doi: 10.1021/acsabm.3c00732 (PMC10792668; doi:10.1021/acsabm.3c00732)
Supplement: Supplementary file 1 — mt3c00732_si_001.pdf [file mt3c00732_si_001.pdf]

Supporting Information

Hydrogel composite magnetic scaffolds: Towards cell free in situ bone tissue engineering

Jingyi Xue[1], Neelam Gurav[1], Sherif Elsharkawy[1], Sanjukta Deb[1*]

[1]Faculty of Dentistry, Oral and Craniofacial Sciences, King's College London, London, United Kingdom, SE1 9RT

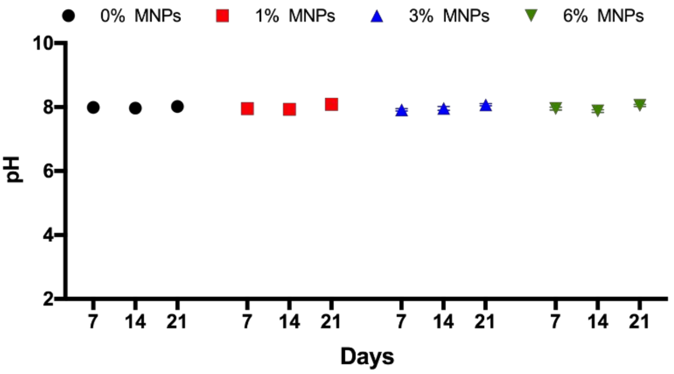

Fig. S1 pH changes during the mineralization study for up to 21 days.

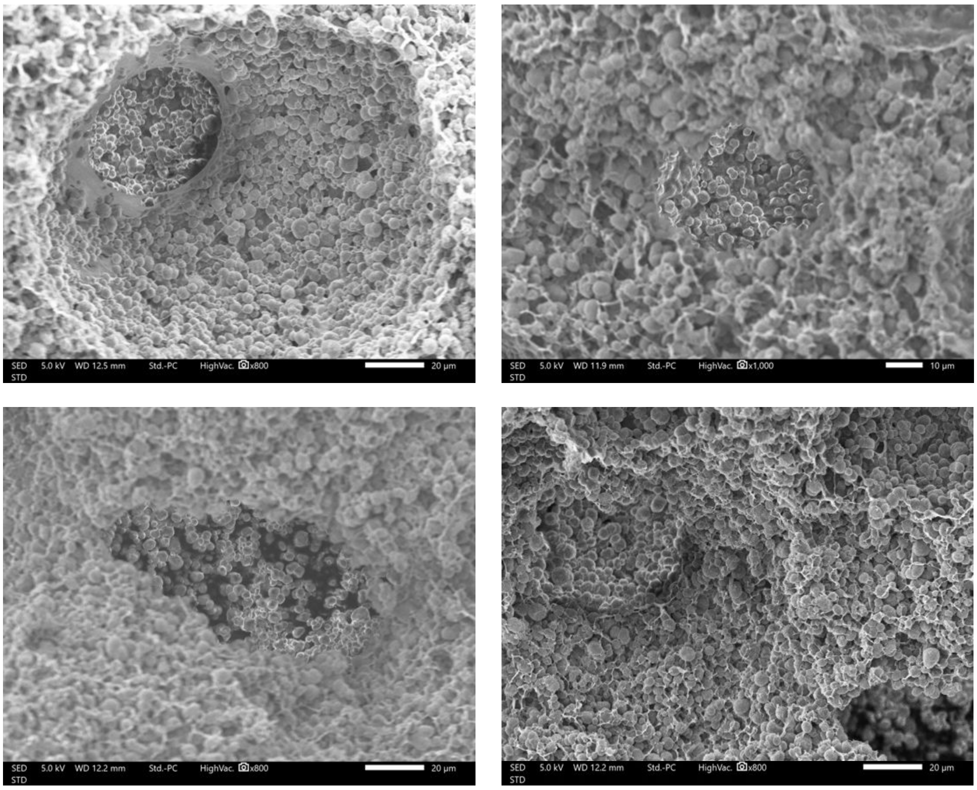

Fig. S2 Representative SEM images of interconnected macropores within the magnetic PVA-Vaterite scaffolds.

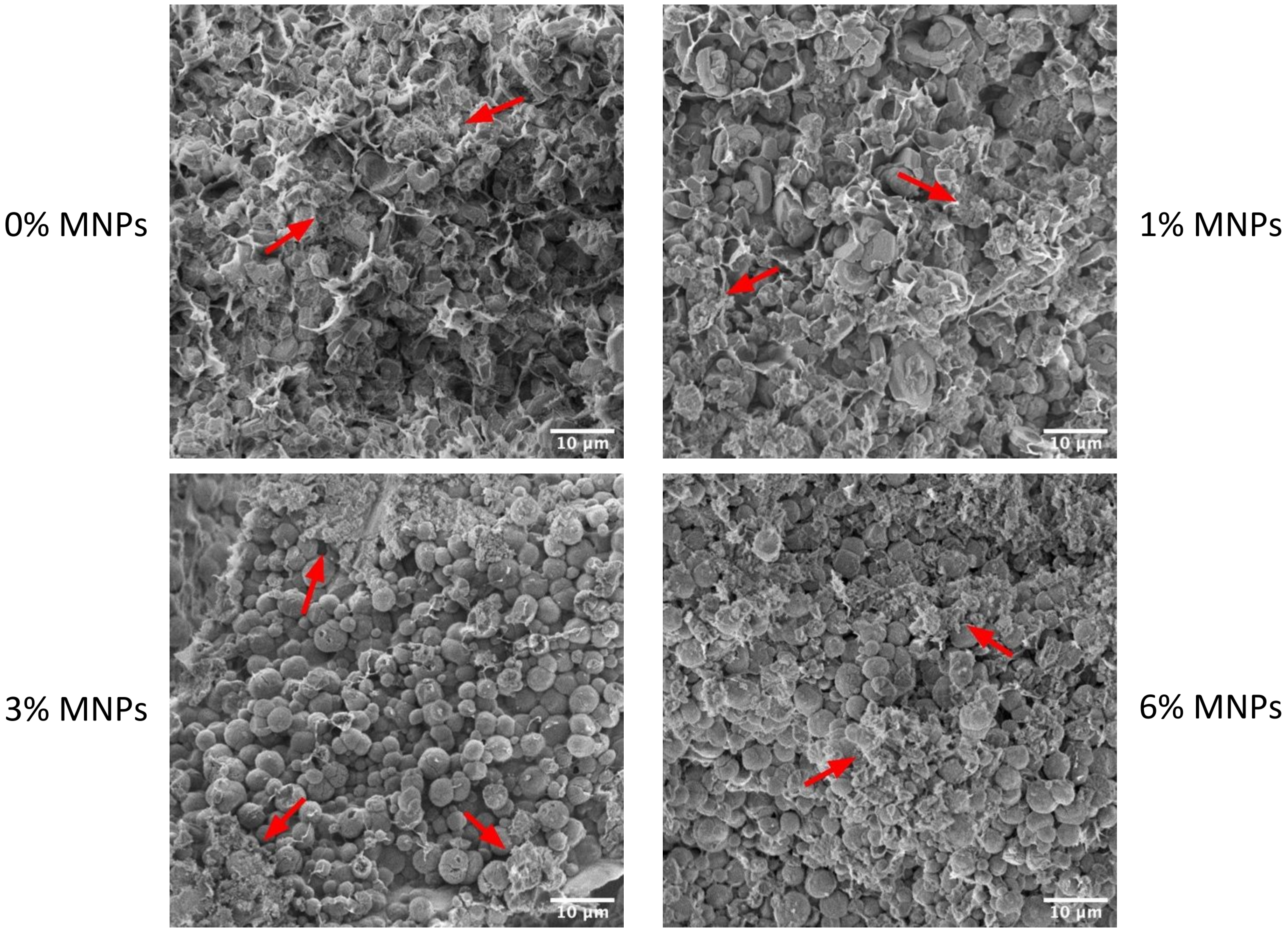

Fig. S3 Cross-section SEM images of magnetic PVA-Vaterite scaffolds after mineralization for 21 days, cauliflower-like apatite crystals were observed within the scaffolds (indicated by red arrows).

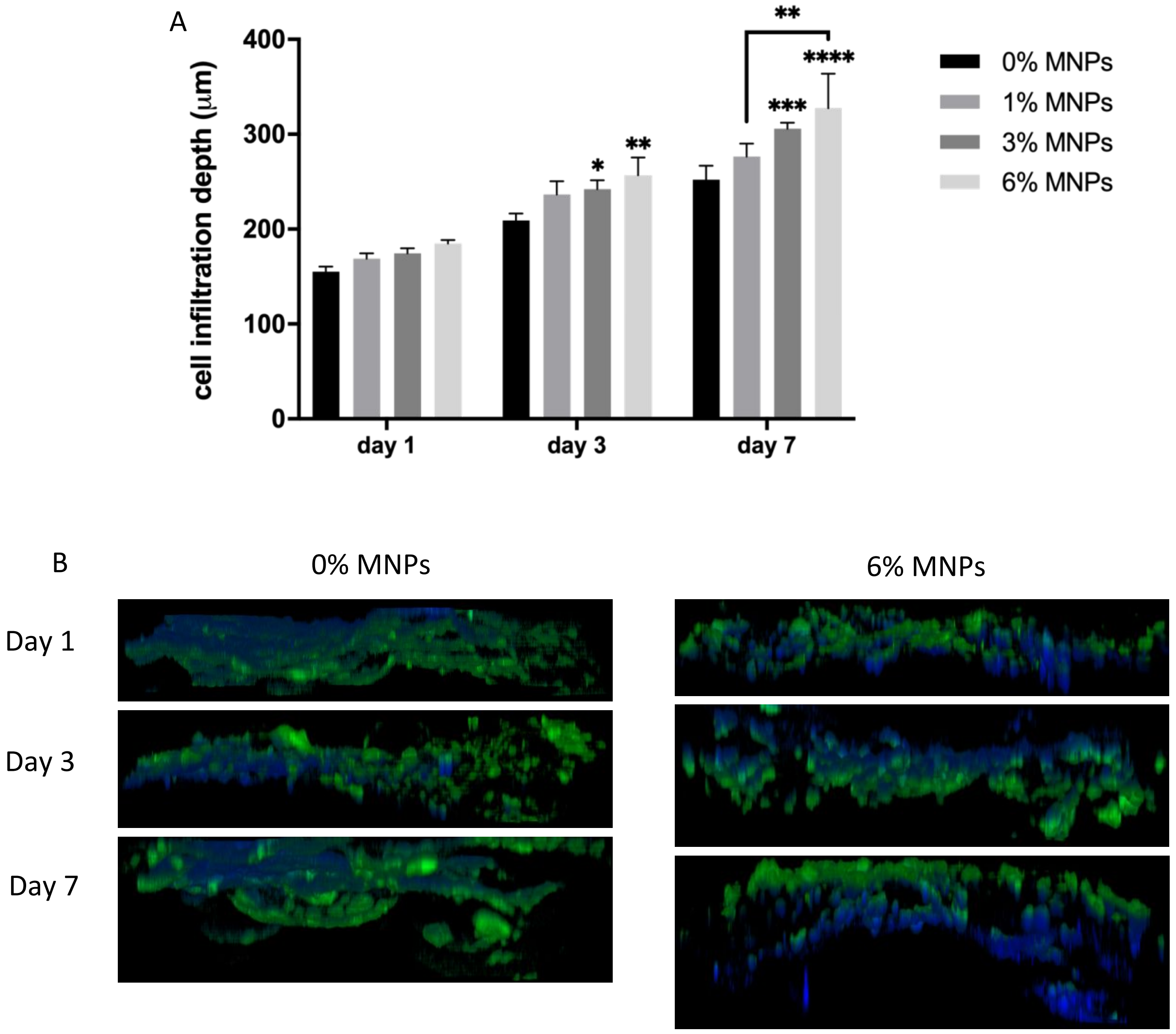

Fig. S4 (A) The quantification of cell infiltration depth, showing improved cell infiltration in the magnetic PVA-Vaterite scaffolds due to enhanced cell attachment and proliferation with the presence of MNPs; (B) representative z-stack images of actin staining of HOS cells (cell skeleton in green and nuclei in blue).
